# Multiple antitumor effects of picropodophyllin in colon carcinoma cell lines: Clinical implications

**DOI:** 10.3892/ijo.2011.1281

**Published:** 2011-12-06

**Authors:** XIAOYING FENG, EIMAN ALEEM, YINGBO LIN, MAGNUS AXELSON, OLLE LARSSON, THOMAS STRÖMBERG

**Affiliations:** 1 Department of Oncology-Pathology, Karolinska Institutet, Cancer Center Karolinska, 17176 Stockholm, Sweden; 2 Department of Gastroenterology, The Second Affiliated Hospital of Dalian Medical University, 116027 Dalian, P.R. China; 3 Faculty of Science, Division of Molecular Biology, Department of Zoology, Alexandria University, 21511 Alexandria, Egypt; 4 Department of Clinical Chemistry, Karolinska Institutet and Karolinska University Hospital, 17176 Stockholm, Sweden

**Keywords:** colon carcinoma, picropodophyllin, insulin-like growth factor-1 receptor, insulin-like growth factor-1, insulin-like growth factor-2, matrix metalloproteinase

## Abstract

Although colorectal cancer can be successfully treated by conventional strategies such as chemo/radiotherapy and surgery, a substantial number of cases, in particular those with liver metastases, remain incurable. Therefore, novel treatment approaches are warranted. The IGF-1R and its ligands, mainly IGF-1 and IGF-2, have been suggested to play pivotal roles in proliferation, survival and migration of adenocarcinoma cells of the colon/rectum. Therefore, interference with IGF-1R-mediated signaling may represent a therapeutic option for this malignancy. In this study, semi-quantitative RT-PCR analyses of 48 paired, colorectal cancer patient samples showed significant overexpression of tumor IGF-1R and IGF-2 mRNA. There was also an overexpression of MMP-7, which was significantly correlated with histopathological parameters. Based on these findings, the effect of the IGF-1R-inhibitory cyclolignan picropodophyllin (PPP) was assessed in the four colon carcinoma cell lines HT-29, HCT-116, DLD-1 and CaCO-2. PPP strongly and dose-dependently inhibited proliferation and migration in all cell lines. However, when exposed to 0.5 μM PPP, only HT-29 showed a net decrease of viable cells as compared with the cell number at the beginning of the experiment, a finding that coincided with decreased expression/phosphorylation of IGF-1R, AKT and ERK. This cell line also exhibited PPP-induced downregulation of MMP-7 and MMP-9. Similar to the DLD-1 and HCT-116 cell lines, HT-29 also showed substantial cell detachment in response to PPP. Although a net reduction of cells by PPP seems to require a synchronized downregulation of IGF-1R, AKT and ERK1/2, part of the antitumor effect may be explained by other, possibly IGF-1R-unrelated mechanism(s). Such a multitude of inhibitory effects of PPP in colon cancer cells together with its low toxicity *in vivo* makes it a promising drug candidate in the treatment of this disease.

## Introduction

Colorectal cancer is the fourth most common cancer in men and the third in women worldwide (1). In countries of Latin America, Asia and Africa, previously exhibiting a low frequency of colorectal cancer, incidence is now rapidly increasing (2), probably due to a rising prevalence of obesity in combination with decreased physical activity and longer life-span (3).

The insulin-like growth factor-1 receptor (IGF-1R) is a transmembrane tyrosine kinase receptor composed of two α subunits and two β subunits. As the ligand (mainly IGF-1 or IGF-2) binds to the IGF-1R α-subunit, tyrosine residues in the intracellular part of the membrane-bound β-subunit become autophosphorylated (4). Subsequent phosphorylation of a chain of intracellular proteins then enables the activation of the phosphatidylinositol 3-kinase (PI3K)/AKT and the mitogen-activated protein kinase (MAPK) pathways leading to proliferation, cell survival and differentiation (5). Several studies have demonstrated increased expression of the IGF-1R in different cancers (6–8), where it facilitates anchorage-independent growth, migration and chemoresistance (9). The cyclolignan picropodophyllin (PPP) has been launched as a potent and selective inhibitor of IGF-1R inhibiting malignant cell growth with low or no toxicity on normal cells (10–14). In preclinical models, PPP exhibits anti-tumor activity in several malignancies, e.g., multiple myeloma, uveal melanoma and glioblastoma (11,14,15) and is currently tested in an open label combined phase I/II clinical study against advanced, solid tumors which have progressed despite several lines of treatment. Encouragingly, stabilized disease has been demonstrated in four PPP-treated squamous non-small cell lung cancer patients (16).

The prognosis of colon cancer after curative-aiming surgery depends almost completely on the presence of metastases, in particular liver metastases (17). The IGF axis seems to play a critical role for the development of these, since colon cancer cells manipulated to express a dominant-negative IGF-1R failed to produce liver metastases although the malignant cells were injected directly into the organ (18). Moreover, increased serum level of IGF-1 in colon cancer patients was demonstrated to correlate with more severe disease (19).

Matrix metalloproteinases (MMPs) play important roles in the process of cancer invasion and metastasis by their capacity to process growth factors, growth factor binding proteins, cell surface proteins and degrade extracellular matrix (ECM) components (20). Mainly MMP-2 (21,22), -7 (23) and -9 (24) have been implicated in colon cancer, where MMP-2, together with MMP-3 and -11, have been suggested to facilitate late-stage tumor invasion and metastasis (21). MMP-7 is widely expressed in colon adenocarcinomas, but has also been proposed to play a role in the early events of tumor progression since low levels have been detected in 50% of benign adenomas (21). Additionally, MMP-7 expression seems to correlate with the metastatic potential of colon cancer cells (23). MMP-9 has been demonstrated in neutrophils and macrophages of the tumor tissue, whereas the tumor cells themselves were negative, suggesting that MMP-9 might be an important part of the inflammatory response elicited by the malignant cells (24).

In this study, we demonstrate a significant overexpression of IGF-1R, IGF-2 and MMP-7 mRNA in colorectal cancer tissues. The treatment of colon cancer cell lines with the IGF-1R inhibitor PPP strongly impaired proliferation and survival, and also negatively affected cell attachment and migration. However, a net decrease of cells seemed to occur only when downregulation of IGF-1R (together with AKT and ERK) expression/phosphorylation was achieved. In addition, PPP showed capability of inhibiting expression of MMP-7 and -9, suggesting multiple targets for this compound, which, together with its good tolerability *in vivo*, might be favorable from a therapeutic point of view.

## Patients and methods

### Patient material

The study subjects comprised 48 histopathologically confirmed colorectal cancer patients who underwent surgery at the Second Affiliated Hospital of Dalian Medical University between June 2005 and February 2006. Patients with previous chemotherapy and radiation therapy, patients with diabetes or hyperthyroidism as well as patients taking glucocorticoids were excluded from the study, which was approved by the local ethics committee according to the Declaration of Helsinki. After informed consent, paired colorectal specimens of tumor and normal control tissues (>10 cm from the tumor) were surgically obtained and placed in liquid nitrogen within 20 min post-excision and processed as described (25).

### RNA isolation and semi-quantitative RT-PCR analysis

Total RNA was extracted from 80–100 mg frozen cancer or normal control tissue from colon/rectum using the TRIzol Reagent (Invitrogen, San Diego, CA) according to instructions provided by the manufacturer. The concentration and the purity of the isolated RNA were analyzed by the spectrophotometer DU-640 (Beckman Coulter, Brea, CA). RNA (1 μg) was used to produce 20 μl of cDNA with TaKaRa RNA LA PCR™ Kit (TaKaRa Biotechnology, Dalian, China). PCR was conducted with TaKaRa Ex Taq HS DNA polymerase in 50 μl reaction volumes. Primers (TaKaRa Biotechnology) used were as follows: IGF-1 (sense 5′-GAAGGTGAAGATGCACACCA-3′, antisense 5′-AGC GAGCTGACTTGGCAGGCTTGA-3′) with a product length of 299 bp, IGF-2 (sense 5′-GGAATCCCAATGGGGAAGT-3′, antisense 5′-TGGGTGGGTAGAGCAATCAGG-3′) with a product length of 488 bp, IGF-1R (sense 5′-ACCCGGAGTACT TCAGAGCT-3′, antisense 5′-CACAGAAGCTTCGTTGAG AA-3′) with a product length of 229 bp, MMP-7 (sense 5′-GAG TGCCAGATGTTGCAGAA-3′, antisense 5′-TGGGGATCTC CATTTCCATA-3′) with a product length of 463 bp and, as internal control, β-actin (sense 5′-GCATGGAGTCCTGTGG CAT-3′, antisense 5′-CTAGAAGCATTTGCGGTGG-3′) with a product length of 326 bp. Using a Thermocycler (Eppendorf, Hamburg, Germany) 35, 35, 35, 32 and 30 PCR cycles were carried out for analysis of IGF-1, IGF-2, IGF-1R, MMP-7 and β-actin mRNA expression, respectively. Each cycle consisted of 30-sec denaturation at 94°C, 40 sec at 63°C, 61°C or 57°C, 30-sec annealing at 59°C or 58°C for IGF-1, IGF-2, IGF-1R, MMP-7 and β-actin primer, and 60-sec extension at 72°C. After amplification, the products were electrophoresed in 2% agarose-1X TAE gels containing 0.5 μg/ml ethidium bromide, the band intensities and the relative ratio between target band and control band were quantified by Labworks Analysis Software (UV, Upland, CA).

### Cell culture

The human colon adenocarcinoma cell lines HT-29, HCT-116, DLD-1 and CaCO-2 were kindly provided by Professor Lars Holmberg and Professor Klas Wiman at CCK, Karolinska Institutet. The cell lines were routinely cultured in complete medium, i.e., DMEM (1 mM pyruvate and 1 g/l D-glucose) supplemented with 10% fetal bovine serum, glutamine and antibiotics (Sigma, St. Louis, MO) at 37°C in a humidified incubator with a 5% CO_2_ in-air atmosphere. All cell lines were confirmed mycoplasma-negative by MycoAlert^®^ (Lonza, Copenhagen, Denmark).

### Analysis of proliferation/viability and surface IGF-1R expression

PPP, synthesized as described (10), was dissolved in DMSO at 10 mM and prediluted in the solvent before addition to cell cultures, where the final concentration of DMSO was always 0.1%. Proliferation/viability was analyzed by using the resazurin assay (14) and by counting using trypan blue exclusion. Surface IGF-1R expression was quantified by flow cytometry using phycoerythrin-conjugated anti-IGF-1Rα and isotype control antibodies (BD Biosciences, San José, CA) as described (14).

### Cell migration analysis

Cell migration was assessed by using the scratch assay (26), where near-confluent cell monolayers were scratched with a plastic micropipette tip, carefully rinsed with PBS and then treated with IGF-1 (R&D Systems, Abingdon, UK) or PPP in complete medium. The degree of closure was photographed at 24 and 48 h through a Plan Fluor 10x/0.30 objective lens (Nikon Instruments Europe BV, Amstelveen, The Netherlands).

### Western blotting

Subconfluent layers of the cell lines were treated with 0.5 μM PPP for different times in complete medium followed by 5-min IGF-1 stimulation before wash in cold PBS and lysis on ice using modified RIPA buffer (50 mM Tris-HCl pH 7.5, 150 mM NaCl, 1 mM NaF, 1 mM EDTA, 1% Igepal CA-630, 0.25% sodium deoxycholate), centrifugation at 14,000 × g for 10 min at 4°C and immunoblotting. Primary antibodies were specific for phospho-IGF-1R (Tyr1135/Tyr1136) (R&D Systems), IGF-1Rβ (C20), GAPDH (Santa Cruz Biotechnology, Santa Cruz, CA), phospho-AKT (Ser473), total-AKT, ERK1/2 (Thr202/Tyr204), total-ERK1/2, MMP-2, MMP-7 and MMP-9 (Cell Signaling Technology, Danvers, MA).

### Statistical analysis

The mRNA data were analyzed statistically by SPSS software version 10.0 (SPSS, Chicago, IL). Student's t-test was used for the analysis of differences in mRNA expression, where P<0.05 were considered statistically significant.

## Results

### IGF-1R, IGF-2 and MMP-7 mRNA are overexpressed in colorectal cancer tissues

**Figure 1:**
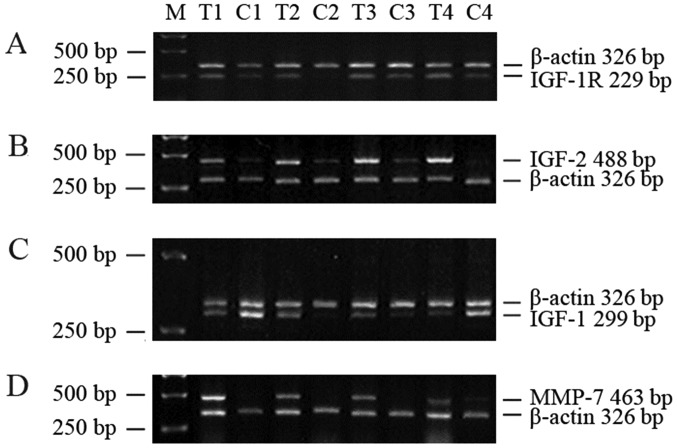
RT-PCR analysis of IGF-1R, IGF-2, IGF-1 and MMP-7 mRNA expression in tissues from colorectal cancer patients. Forty-eight matched samples from tumor (T) and normal control (C) tissue were analyzed for target gene expression by semi-quantitative RT-PCR analysis, where expression of β-actin mRNA served as internal reference. Results from four representative patients are shown.

Tumor IGF-1R and IGF-2 mRNA expression were clearly elevated in 48 paired samples (Table I) as demonstrated in tissues from four representative patients (Fig. 1A and B). However, there was no significant difference in IGF-1 mRNA expression between tumor and normal control tissue (Table 1 and Fig. 1C). MMP-7 mRNA was present in tumor tissues, but could not be detected in normal control tissues (Fig. 1D). Analysis of the relationship between MMP-7 mRNA expression and histopathological parameters demonstrated a significant correlation with depth of infiltration and lymph node metastasis (Table II). No correlation was found between MMP-7 and tumor size.

### PPP reduces proliferation and survival in human colon cancer cell lines

**Figure 2:**
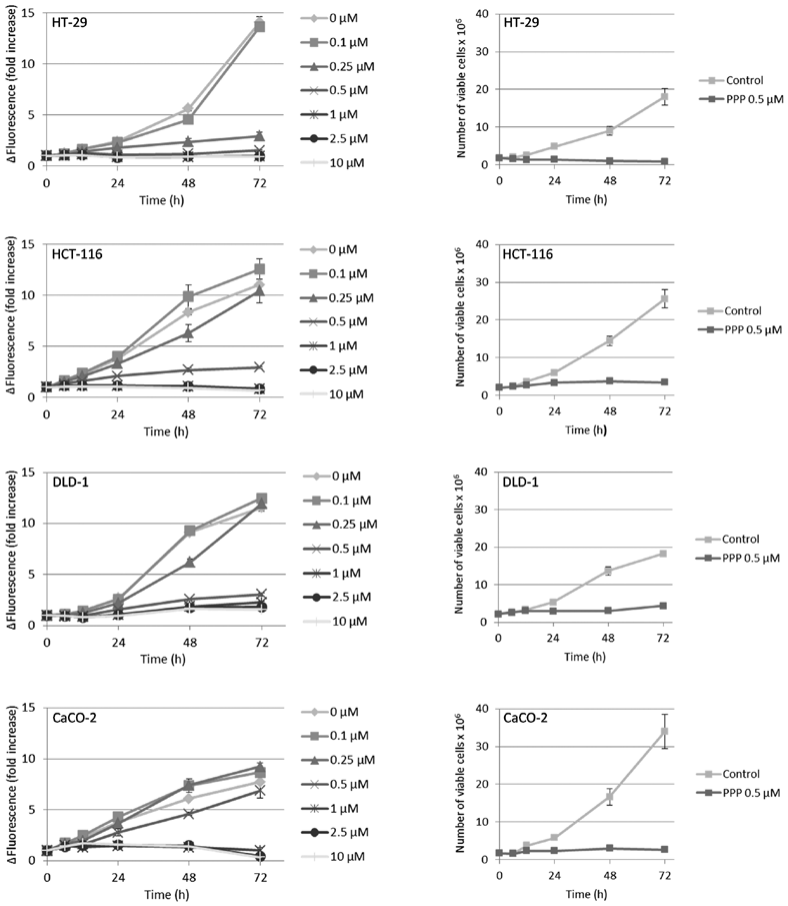
Effect of PPP on growth of colon cancer cell lines. Left panel, cell lines were treated with different concentrations of PPP in triplicates followed by analysis using the resazurin assay at indicated times. Right panel, in parallel experiments, cells were treated with 0.5 μM PPP and counted manually in quadruplicates using trypan blue exclusion resulting in total number of viable cells treated and non-treated with PPP. Three experiments were performed and one representative is shown exhibiting means ± SD.

**Figure 3:**
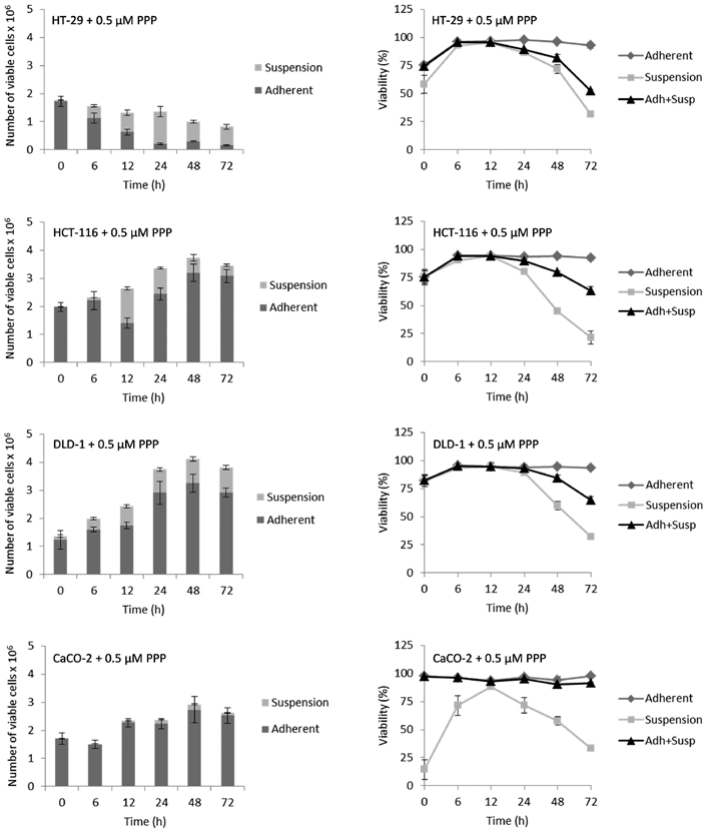
Effect of PPP on cell viability. Left panel, cells were treated with 0.5 μM PPP and counted manually in quadruplicates using trypan blue exclusion resulting in number of viable adherent and suspension cells, respectively. Right panel, in parallel, the number of dead adherent and suspension cells was counted and viability calculated and expressed as mean % viable cells ± SD. All experiments were performed three times and one representative is shown.

The results from the resazurin assay indicated that all cell lines except the DLD-1, when compared with the fluorescence at the beginning of the experiment (0 h), exhibited a net reduction of viable cells when treated with PPP at concentrations ≥1 μM (Fig. 2, left panel). However, since the resazurin assay only provides a relative estimation of viable cell number, we also counted cells by trypan blue exclusion, then using the intermediate PPP concentration 0.5 μM. Furthermore, adherent and suspension cells were collected and counted separately. In these experiments, only the HT-29 cell line exhibited a net reduction of viable cells as compared with the cell number at the beginning of the experiment (Fig. 2, right panel). The HCT-116, DLD-1 and CaCO-2 cell lines continued to proliferate, although at a lower rate, reaching a plateau at 48–72 h. Moreover, the HT-29, HCT-116 and DLD-1 cell lines demonstrated a substantial detachment of viable cells in response to PPP, an effect that occurred already at 6–12 h (Fig. 3, left panel) and in parallel with reduced proliferation (Fig. 2, right panel). The vast majority of the detached, viable cells were accumulated in the G_2_/M-phase of the cell cycle (data not shown). With the exception of the CaCO-2 cell line, there was a general, time-dependent decline in cell viability, an effect that was exclusively due to dying suspension cells (Fig. 3, right panel). However, cells that despite PPP-treatment remained attached retained high viability (Fig. 3, right panel). The degree of growth inhibition induced by 0.5 μM PPP (Fig. 2, right panel) exhibited a linear correlation to the cell line population doubling times (Table III) of HCT-116, DLD-1 and CaCO-2 (r^2^=0.94). The expression of surface IGF-1R was highly variable, i.e. CaCO-2 cells expressed nearly ten times more IGF-1R than the DLD-1 cells, whereas the HT-29 and HCT-116 cell lines showed intermediate levels (Table III).

### PPP inhibits cell migration

We then examined the effects of PPP on migration by using the scratch assay (26). As compared with control and IGF-1 treated cells, scratch closure was clearly reduced by PPP in a dose-dependent manner in all four cell lines (Fig. 3), this effect being least pronounced in the CaCO-2 cells.

### PPP downregulates phosphorylation/expression of IGF-1R, AKT and ERK in HT-29 cells

**Figure 4:**
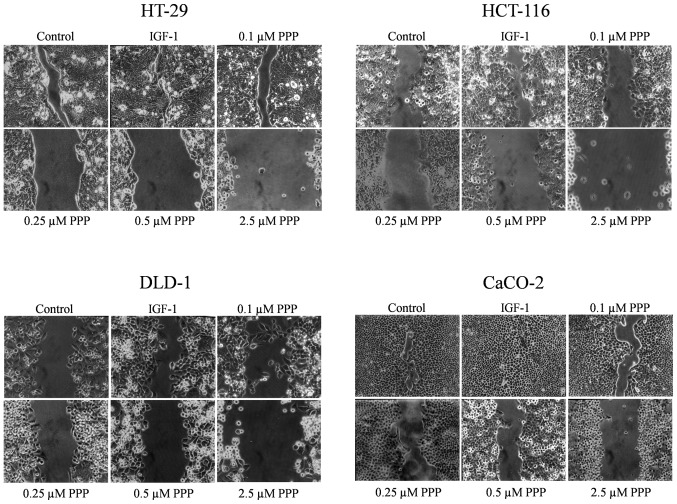
Effects of IGF-1 and PPP on cell migration. Petri dishes with near-confluent monolayers of the cell lines were scratched before treatment with 50 ng/ml IGF-1 or indicated concentrations of PPP for 48 h (HT-29) or 24 h (HCT-116, DLD-1 and CaCO-2). Three experiments were performed and images show representative areas of quadruplicate scratches.

Next, the effects of PPP on IGF-1R, AKT and ERK and their phosphorylated forms were examined. As expected, treatment of HT-29 and HCT-116 cells with IGF-1 increased phosphorylation of the IGF-1R, AKT and ERK (Fig. 4A). This was also true for the DLD-1 and CaCO-2 cell lines (data not shown). Pretreatment of HT-29 cells with 0.5 μM PPP decreased the IGF-1-induced phosphorylation of IGF-1R, AKT and ERK, effects that were time-dependent starting at 8 h for IGF-1R and AKT and at 12 h for ERK (Fig. 4A, left panel). In HCT-116 cells, only AKT phosphorylation was reduced (Fig. 4A, right panel). Moreover, this effect occurred at a later time-point (12 h) than in the HT-29 cells. Total-IGF-1R showed a marked, time-dependent downregulation in the HT-29 cell line beginning already at 4-h treatment with PPP. Such a response could not be detected in the HCT-116 cell line (Fig. 4A). In addition, total-AKT and total-ERK was downregulated in the HT-29 cells at 12 and 24 h, effects that could not be clearly established in the HCT-116 cell line (Fig. 4A). The DLD-1 and CaCO-2 cells showed no downregulation of phosphorylated or total forms of IGF-1R, AKT and ERK in response to PPP (data not shown).

### PPP reduces the expression of MMP-7 and MMP-9

Since MMP-2, -7 and -9 have been associated with different aspects of colon cancer (21–24), we assessed whether IGF-1 and PPP could affect their protein expression in the HT-29 cell line. IGF-1 increased the expression of MMP-7, whereas pretreatment with 0.5 μM PPP for 8 h inhibited this effect (Fig. 4B). IGF-1 was unable to affect the expression of MMP-9, however, when combined with PPP, MMP-9 was downregulated. MMP-2 expression showed no regulation in response to IGF-1 alone or in combination with PPP (Fig. 4B).

## Discussion

Accumulated evidence during the last decades indicate a pivotal role for the IGF-1R signaling in cancer cells, suggesting this receptor to be a promising molecular target in cancer treatment (27). However, very few attempts have been made to interfere with its function in colon cancer (18,28). Therefore, in parallel to analyses of IGF-1R, IGF-1/2 and MMP-7 mRNA expression in tissue from colorectal cancer patients, we assessed the effects of the IGF-1R-inhibitory compound PPP in four colon carcinoma cell lines.

Tumor tissue extracted from 48 colon cancer patients significantly overexpressed IGF-1R and IGF-2 mRNA, but not IGF-1 mRNA. These findings are basically in accordance with previous studies (29–32), thus strongly emphasizing the potential impact of the IGF axis in the development and progression of colon cancer. The expression of MMP-7 mRNA in all colon tumors and its correlation with depth of invasion/serosal involvement and lymph node metastasis were as well confirmatory (21,23). Besides that elevated MMP-7 expression seems to correlate with the metastatic potential of colon cancer, it should be noted that the capability of this MMP to locally increase the IGF-1/2 bioavailability by cleavage of IGF-1 binding protein(s) (32) might contribute to increased proliferation/survival and metastatic potential of the malignant cells.

Multiple approaches have been utilized to block IGF-1R signaling *in vitro* and *in vivo*. We used the small molecule inhibitor PPP, which inhibited proliferation/survival of the four colon cancer cell lines in a dose-dependent manner as determined by the resazurin assay. However, the DLD-1 cell line was the only one that did not exhibit a net decrease of relative number of viable cells although it was treated with 10 μM PPP as analyzed by the resazurin assay. In contrast, the other cell lines showed diminished relative cell number when treated with ≥1 μM PPP. A more detailed investigation by manual cell counting, here exposing the cells to 0.5 μM PPP revealed that all cell lines except the HT-29, despite PPP-treatment, continued to slowly proliferate until they reached a plateau. The HT-29 cell line instead showed a net decrease of viable cells as compared with the cell number at the beginning of the experiment. This finding is also supported by the linear correlation between the population doubling times and PPP-mediated growth inhibition in the colon cancer cell lines that could be established only when the HT-29 cell line was omitted. Interestingly, only in this cell line 0.5 μM PPP was able to downregulate the IGF-1R, which would fit with the hypothesis that the IGF-1R has to be downregulated/degraded for extensive tumor cell kill (33,34). Downregulation of IGF-1R in the HT-29 cell line seemed to precede PPP-mediated inhibition of IGF-1-induced phosphorylation of IGF-1R and AKT/ERK. Although these findings differ from some other investigations (10,35), a similar PPP-induced downregulation of both IGF-1R/AKT and their phosphorylated forms has been shown in a multi-drug resistant osteosarcoma cell line (36). The relatively late downregulation of ERK1/2 and its phosphorylated form in the HT-29 cell line, however, represents a novel finding as compared with published results obtained using other cell lines (10,34). A similar, more or less simultaneous downregulation of various intracellular signaling proteins along with their phosphorylated forms has also been observed in multiple myeloma cell lines after 48 h of exposure to PPP (unpublished data). However, the explanation behind this phenomenon and its potential clinical relevance for anti-cancer treatment remains elusive.

Inhibition of proliferation in the colon carcinoma cell lines was detected already at 6–12 h of PPP-incubation and paralleled detachment of viable cells in the cell lines. In contrast, PPP-induced cell death occurred later (~24 h) and exclusively affected the detached cell population. Thus, the CaCO-2 cells, exhibiting only small amounts of detached cells, showed pronounced decrease in proliferation but negligible cell death when exposed to 0.5 μM PPP. Possibly, the high expression of IGF-1R in this cell line might provide survival advantage(s) as suggested for glioblastoma cell lines (14).

Using the scratch assay (26), we investigated colon cancer cell migration during PPP-treatment. Migration was dose-dependently inhibited, suggesting that PPP might reduce invasion as well as the process of metastasis of colon cancer cells. Since this effect, which has not been reported previously, was evident in all cell lines, we propose mechanism(s) unrelated to signaling via IGF-1R/AKT/ERK as responsible. In this context, it is interesting to note that we could not detect PPP-mediated inhibition of MMP-2 expression as previously shown in uveal melanoma cell lines (15). Instead, we demonstrated downregulation of MMP-7 and -9.

In conclusion, PPP strongly decreases proliferation, survival and migration and also promotes detachment in colon cancer cell lines. Although a net reduction of tumor cells seems to correlate with PPP-induced downregulation of IGF-1R, AKT and ERK, the effects described above, including the downregulation of MMP-7 and -9, may partly be independent of IGF-1R inhibition. Further investigations are required to disclose such, possibly IGF-1R unrelated mechanism(s). However, the multiple inhibitory effects of PPP in colon carcinoma cells combined with the promising results from the open label combined phase I/II clinical study against advanced solid tumors (16), suggest a rationale for the therapeutic use of PPP in colorectal carcinoma.

## Figures and Tables

**Figure 5:**
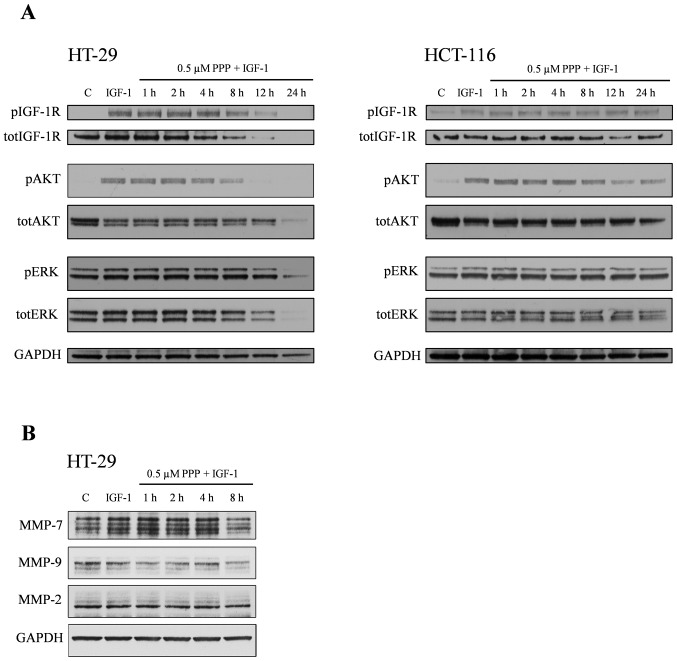
Effects of PPP and/or IGF-1 on expression of AKT, ERK and MMP-7, -9 and -2. The cell lines were pretreated for indicated times with 0.5 μM PPP in complete medium and then stimulated 5 min with 50 ng/ml IGF-1 before lysis. (A) The expression of phospho/total-IGF-1R, phospho/total-AKT and phospho/total-ERK in the HT-29 and HCT-116 cell lines and (B) MMP-7, -9 and -2 in the HT-29 cell line were analyzed by Western blotting where GAPDH served as loading control. All experiments were performed three times and one representative is shown.

**Table I:** IGF-1R, IGF-2 and IGF-1 mRNA expression in tumor (T) and normal control (C) tissue of 48 colorectal carcinoma patients.^a^

mRNA	T (mRNA/β-actin)	C (mRNA/β-actin)	P-value
IGF-1R	0.607±0.327	0.333±0.22	0.027
IGF-2	0.979±0.436	0.293±0.261	0.000
IGF-1	0.529±0.546	0.329±0.194	0.246

a Data are presented as means ± SD.

**Table II:** MMP-7 mRNA expression in tumor tissue and histopathological characteristics of 48 colorectal carcinoma patients.^a^

Parameter	No. of cases	MMP-7 mRNA/β-actin	P-value
Serosal involvement			
With	29	1.310±0.402	
Without	19	0.872±0.376	0.030
Lymph node metastasis			
With	27	1.390±0.381	
Without	21	0.942±0.376	0.010
Tumor size (cm)			
≥5	31	1.332±0.361	
<5	17	1.019±0.469	0.190

a Data are presented as means ± SD.

**Table III:** IGF-1R expression and population doubling times of colon carcinoma cell lines.^a^

	HT-29	HCT-116	DLD-1	CaCO-2
Population doubling time (h)	37–45	30–32	36–42	22–24
Surface IGF-1Rα expression (RFI)	11.1±1.3	5.7±0.3	3.3±0.6	30.0±5.9

a Population doubling times were calculated from exponentially growing cells by manual cell counting. Relative cell surface IGF-1Rα expression in intact cells was analyzed by flow cytometry in three independent experiments and expressed as mean relative fluorescence intensity (RFI) ± SD.
